# Traditional knowledge of wild food plants in a few Tibetan communities

**DOI:** 10.1186/1746-4269-10-75

**Published:** 2014-11-03

**Authors:** Alessandro Boesi

**Affiliations:** Via Luigi Zoja 1, 20153 Milan, Italy

**Keywords:** Wild edible plants, Traditional knowledge, Tibet

## Abstract

**Background:**

This paper aims to present the author’s field research data on wild food plant use in Tibetan regions. It provides a general perspective on their significance in past and present Tibet, and examines the concept of wild edible plants as medicinal plants. The fieldwork was conducted in Dhorpatan (Nepal, May-August 1998), Lithang town and surroundings (Sichuan, China, April-September 1999, May-August 2000); Southern Mustang District (Nepal, July-August 2001); and Sapi (Ladakh, Jammu and Kashmir, India, July 1995, August 2005).

**Methods:**

The research was conducted with 176 informants. The methodology included ethnographic research techniques: participant observation, open-ended conversations, semi-structured interviews, and studies of Tibetan medical texts. The author worked in the field with Tibetan colloquial and written language.

**Results:**

The 75 total wild food plants and mushrooms belong to 36 genera and 60 species. 44 specimens are used as vegetables, 10 as spices\condiments, 15 as fruits, 3 as ferments to prepare yoghurt and beer, 5 as substitutes for *tsampa* (roasted barley flour, the traditional staple food of Tibetan people), 4 as substitutes for tea, and 3 to prepare other beverages. Data from Lithang, which are more representative, show that among 30 wild food plant species exploited, 21 are consumed as vegetables, 5 as spices, 4 as fruits, 3 represent substitutes for roasted barley flour, 2 substitutes for tea, and 1 is used as fermentation agent.

**Conclusion:**

Tibetans have traditionally exploited few wild food plants. These mainly compensate for the lack of vegetables and fruit in traditional Tibetan diet, notably among pastoralists, and are far more important during famines as substitutes for roasted barley flour. Today few wild food plants are regularly consumed, less in the main towns and villages and moreso in remote areas and among pastoralists. Younger generations from towns have almost lost traditional botanical knowledge. Owing to modernisation and globalisation processes, many local people have specialised in collecting natural products increasingly demanded in China and abroad. Tibetan people strongly benefit from these activities. Tibetan medicine sees diet as a way of curing diseases and medical treatises describe therapeutic properties of several wild food plants that Tibetans nowadays consume.

## Background

### Introduction

Tibetan peoples traditionally live over a vast area located in central Asia almost corresponding to the land mass occupied by the Tibetan plateau. As Kapstein [1] states: “In its socio-economic dimensions Tibet may be thought of in terms of its dominant forms of production: high-altitude pastoralism and a barley-based agriculture. Culturally Tibet is distinguished by the use of classical Tibetan as a literary medium, by shared artistic and craft traditions, and by the important role of the religious system of Tibetan Buddhism. Politically, according to one’s ideological standpoint or historical frame of reference, Tibet may be a particular administrative unit of the contemporary People’s Republic of China, the Tibet Autonomous Region, or else the much vaster territory…” inhabited by Tibetan cultural people, including other Chinese administrative units (Northern Yunnan, Western Sichuan, Western Gansu, and Qinghai Provinces), and regions located in the western portion of the Tibetan plateau and astride the Himalayan Range, which nowadays belong to India, Nepal, and Bhutan.

Up to the 1950s, when the Chinese Liberation Army occupied it, Tibet has remained almost isolated - both culturally and politically - from the civilizations that flourished in the surrounding countries. Also the other regions belonging to the Tibetan world and not included in today’s Tibetan areas within the PRC have remained for a long time without any significant influence from the external world and only in the last few decades have started to be part of modernisation and globalisation processes. This isolation allowed Tibetan traditional culture to be preserved to foreign influences, and notably from western influence imposed by colonial powers in many Asian countries in the past centuries, except for the Younghusband Expedition into Tibet in 1904.

Since the Tibetan plateau was first inhabited by humans some 6,000-7,000 years ago, as a variety of research suggests [1], context-specific knowledge of local environment has developed, concerning for example climate, soil features, wild animals, vegetation, plant qualities and use. This expertise originated from the constant relationship between humans, plants, animals, and natural phenomena, and also from religious concepts both autochthonous and imported from foreign lands, particularly India [2, 3], from the 7^th^ century onwards, when Tibetan civilization as we know it now started to develop.

The Chinese takeover of Tibet gave way to tremendous political, economic, and cultural transformations that significantly changed the life of local people. The continued socio-political and economic instability and constraint, and, particularly in the last few decades, the effects of the globalized market system have been affecting Tibetan traditional knowledge of the natural world, and have significantly changed the extent and ways of its exploitation. For example during the Cultural Revolution the intent of revolutionary activity in Tibet was essentially to “destroy the social and cultural fabric of Tibet’s traditional way of life” [4], and Tibetan traditional medicine with its knowledge about medicinal plants was defined as one of the “four olds”: old ideas, culture, customs, and habits. Tibetan doctors were forbidden to practise medicine and large numbers of medical treatises were destroyed [5].

### Interactions between people and wild plants in the Tibetan world

Wild plants have always represented an important resource for Tibetan populations [6], notably for cattle breeding, house construction, tool manufacture, as a source of fuel (mainly in the form of yak dung), dying materials, and perfumes. For more than a millennium Tibetan medical practitioners have been relying, to prepare their remedies, on many plants growing in the wild, several of which have also been collected and traded by professional and occasional dealers. In meadows and pastures Tibetan children play with flowers. People place on altars in houses and temples flowers collected in the wild. Greatly appreciated for their beauty and fragrance as offerings to divinities, plants are also used in religious ceremonies. Tibetan medical texts describe the miraculous creation of certain plants through the intervention of divinities and religious personages [7]. And, last but not least, wild plants have been collected to be consumed as food, the subject matter of this article.

Travel accounts dated to pre-modern Tibetan regions [8–12], research data [13–18], and the author’s fieldwork data [2, 6, 19], show that in some Tibetan activities the use of wild plants is less important today than it was in the past whereas in others it is more important now. Actually in the past few decades the gathering and trading of natural substances has even become a crucial activity for the economy of whole towns and villages.

Political, social, economic, and cultural transformations occurring in Chinese Tibetan regions since the 1950s have changed Tibetan people’s way of life. Various modern products coming from adjacent and distant regions can be easily purchased in most Tibetan towns and villages, among them tools and items that in pre-modern Tibet people themselves used to manufacture with materials taken from the natural environment. This trend occurs also outside China in Indian and Nepalese areas inhabited by Tibetan populations.

Modernisation processes, notably road improvement, result in natural products being transported quickly from remote areas to trading points. So several wild plant and mushroom species having economic value as medicine and food, and the demand of which is growing in regional, national, and international markets, have been increasingly collected in the last few decades. The revenues from this business nowadays represent an important percentage of the total annual income for several Tibetan communities. The collection and trade of the well-known *yartsa gunbu* (*Ophiocordyceps sinensis*) is the best example of this phenomenon [17–20].

### Aims of the article

This article aims at presenting the field research data on the use of wild food plants, which the author collected in several Tibetan cultural regions, and at trying to provide a general perspective on their significance in Tibetan past and present life and culture. The issues related to the use of wild food plants in the local diet as vegetables, fruits, spices, in the preparation of beverages, as agents in fermentation processes, are addressed, and wild food plants consumed in case of famine, by Tibetan hermits, and their concept and significance in Tibetan medicine, are examined. A brief section devoted to examining local concepts related to the nature of edible mushrooms is included. The author’s field data are possibly not exhaustive for three of the four study regions, yet include the most important wild foods plants that Tibetan people know and use.

### State of the art

Specific in-depth research into wild food plant use among Tibetan populations living in Chinese Tibetan regions is rare [21, 22]. In the Tibetan cultural regions located outside China a few ethnobotanical surveys examine wild food plants and a few anthropological studies mention the use of wild plants in the local diet. Bhattarai and Chaudhary [23, 24] investigate the wild food plants in Manang and Mustang (Nepal), particularly focusing on the ethnobotany of wild *Allium* and *Rosa* species in Manang [25, 26]. Lama and Ghimire [27] report some information on the use of wild plants in local diet in their book devoted to medicinal plants of Dolpo (north-west Nepal). Polhe [15] investigates the useful plants of Manang District, and provides a list of the nutritive plants used in the region. Jest [11] briefly examines the use of wild food plants in Dolpo (north-West Nepal). Sacherer [14], who investigates the ethnobotany of the Rolwaling Sherpa in east Nepal, devotes a paragraph of her article to edible plants. Meyer [28] examines the nutritional practice and medical dietetics in traditional Tibetan medicine. Also, some scholars [12], travellers and missionaries [8–10] living in and exploring Tibetan regions before the 1950, in what may be defined as pre-modern Tibet, occasionally report the gathering and use of certain plants as food.

## Methods

In the context of a doctoral research on the Tibetan concept of plants and the *materia medica* of Tibetan medicine, and additional study (1995, 2005), the author conducted research fieldwork in a few regions inhabited by Tibetan people: Dhorpatan, May-August 1998 (Baglung District, Nepal); Lithang town and surrounding area, April-September 1999 and May-August 2000 (Lithang County, Sichuan, China); Southern Mustang District, particularly Jarkhot village, July-August 2001 (Nepal); and Sapi (some data were also collected in Leh town, Kortshog and Kanji villages), July 1995 and August 2005 (Ladakh, Jammu and Kashmir, India). The author also interviewed people in Shar Khumbu, but did not collect plant specimens there, in August 1998 (Solukhumbu District, Nepal). The research aimed at studying plant ontology, identification, nomenclature, classification criteria, and ways of exploitation among Tibetan people, as well as examining the nature of Tibetan *materia medica* from the gathering of medicinal substances to their categorization and attribution of therapeutic properties. During fieldwork the author collected information on the present use and knowledge of wild food plants, which are presented in this article. The research was conducted with 176 educated and non-educated informants among villagers, pastoralists, Tibetan doctors, and Buddhist monks, including 109 female (62%), and 67 males (48%). Informants’ age spans from 10 to 69 among pastoralists, and from 26 to 74 among settled people. Methodology was based on ethnographic research techniques primarily including participant observation, open-ended conversations, semi-structured interviews, and studies of local medical texts (they sometimes include information on wild food plants). The data were primarily obtained by observing wild food plant collection and use during the long time that the author spent with Tibetans, and by using semi-structured interviews. The key questions concerned plant local names, place of growth, time and frequency of collection, part used, reason, mode, and frequency of use at present and in the past, presence of varieties, necessity of plant transformation and purification processes.

The author specifically worked with colloquial Tibetan and written Tibetan language. The knowledge of Tibetan language and the long time spent in the field helped to establish friendly relations with people and to benefit from the full cooperation and consent of informants, independently from their age, gender, and social status. The phonetic transcription of Tibetan names is italicised, their proper Tibetan spelling is given in Tables 1, 2, 3, and 4, according to the Wylie [29] system of transliteration (minus the hyphen in between syllables).

**Table 1:** **The wild food plants collected in the study areas**

Plants collected in Sapi, Ladakh, Jammu and Kashmir, India				
Botanical identification	Tibetan name	Part used	Use in local diet	C. index*
*Arnebia euchroma* (Royle ex Benth.) I.M. Johnston	*‘bri mog*	roots	used as spice to cook meat	+++
*Artemisia gmelinii* Weber ex Steckm. var. *gmelinii*	*bur tse, mkhan pa,*	leaves, flowers	mixed with wheat flour and water to prepare ferments	++++
*Capparis spinosa* L.	*kabra*	fruits	unripe fruits eaten as vegetables	++++
*Chenopodium album* L.	*sne’u*	leaves	stir-fried in oil after eliminating their bitter taste by boiling them long time in water, and are eaten with other food	++++
*Delphinium brunonianum* Royle	*bya rgod spos*	leaves, flowers	mixed with wheat flour and water to prepare ferments	+++
*Hippophae rhamnoides* L. subs. *turkestanica* Rousi	*tshogs skyur, star bu*	fruits	fruits eaten mainly in the past, today a juice is industrially prepared from them	+++++
*Oxyria digyna* Hill	*chu lcum*	leaves	eaten fresh as vegetable	+++
*Rheum spiciforme* Royle	*chu rtsa*	stems	petioles and young stems eaten as vegetables	+++++
*Rosa sericea* Lindl.	*se ba*	fruits	eaten by children	++++
*Rosa webbiana* Wallich ex Royle	*se ba*	fruits	eaten by children, used to prepare a kind of jam	+++++
*Thymus linearis* Benth.	*su lu*	leaves and stems	mixed with chilly are used as condiment	+++++
*Urtica hyperborea* Jacquem. ex Wedd.	*zwa*	young shoots	young shoots used to prepare soups	+++++
**Plants collected in Lithang County, Sichuan, China**				
*Allium macranthum* Baker	*byi’u sgog*	bulbs	eaten fresh as vegetable and spice	+++++
*Allium prattii* C.H. Wright	*rug sgog*	bulbs	eaten fresh as vegetable and spice	+++++
*Allium* sp.	*sha sgog*	bulbs	eaten fresh as vegetable and spice	++++
*Allium* sp.	*sgog pa*	bulbs	eaten fresh as vegetable and spice	+++++
*Arisaema flavum* (Forssk.) Schott.	*dwa ba, dwa g.yung*	tubers	eaten after being crushed and boiled	++++
*Berberis* sp.	*skyer pa*	fruits	eaten by children	+++
*Capsella bursa-pastoris* (L.) Med.	*sog ka pa*	leaves	fresh leaves are fried with vegetables, dry leaves are eaten in local soups (*thug pa*)	+++++
*Carum carvi* L.	*go snyod*	seeds	the crushed seeds are used as a spice	+++++
*Chenopodium album* L.	*sne’u*	leaves	stir-fried in oil after eliminating their bitter taste by boiling them long time in water, and are eaten with other food	++++
*Cirsium souliei* (Franch.) Mattf.	*spyang tsher*	roots	eaten raw after removing the skin	++
*Cynanchum* sp.	*dug mo nyung*, *pha la*	roots	in the past eaten boiled	++
*Galium aparine* L.	*zangs rtsi dkar po, phyi ‘dzin pa*	stalks, leaves	stalks and leaves rubbed between hands are used as fermentation agent in the making of yoghurt.	+++
*Lepidium apetalum* Willd.	*dar ya kan, khang phug*	leaves	the leaves are cooked in water	++++
*Malva verticillata* L.		leaves	stir-fried in oil before adding other vegetables and/or meat	++++
*Plantago depressa* Willd.	*tha ram*	leaves	leaves eaten as vegetable	++++
*Sinopodophyllum hexandrum* (Royle) T. S. Ying	*‘ol mo se, ba ma lu lu*	fruits	children eat fresh fruits	++++
*Polygonum macrophyllum* D.Don	*spang ram*	roots, seeds	roots eaten fresh, flour obtained from ground seeds used as substitute for *tsampa* in the past	++++
*Polygonum polystachyum* Wallich ex Meisner	*snya lo*	stems	stems eaten raw after removing the skin	++++
*Polygonum viviparum* L.	*ram bu rgod pa*	roots, seeds	roots eaten fresh, flour obtained from ground seeds used as substitute for *tsampa* in the past	+++++
*Potentilla anserina* L.	*gro ma*	rhizomes	rhizomes eaten fresh and cooked, also during famines in the past	+++++
*Potentilla* sp.	*ston ja*	aerial portion	in the past used as a substitute for tea	++++
*Quercus* sp.	*be do shing*	acorns	flour obtained from dry acorns used in the past as substitute for *tsampa* (roasted barley flour)	+++++
*Rheum alexandrae* Batal.	*chu skyur*	stems	stems eaten raw after removing the skin	+++++
*Rheum palmatum* L.	*lcum, shog sbra*	stems	stems eaten raw after removing the skin	+++++
*Rhododendron* sp.	*sur dkar*	flowers and leaves	in the past used as substitute for tea	+++++
*Rosa omeiensis* Rolfe	*se ba*	fruits	fruit edible (today eaten by children)	++++
*Rubus subornatus* Focke	*stag tsher*	fruits	eaten fresh	++
*Taraxacum officinale* L. *s.l.*	*khur mang, khur dkar, khur nag, nyin dgun me tog, rnag gi me tog*	leaves	the leaves are fried in oil or cooked in water	+++++
*Thlaspi arvense* L.	*bre ga, ‘dre rnga*	leaves	fresh leaves are fried with vegetables, dry leaves are eaten in local soups (*thug pa*)	+++++
*Urtica triangularis* Hand. - Mazz.	*zwa*	young shoots	used to prepare soups	+++++
**Plants collected in southern Mustang District, Nepal**				
*Allium roseum* L.	*‘dzim bu*	bulbs	eaten fresh as vegetable and spice, kept to be consumed in winter	+++++
*Arisaema flavum* (Forssk.) Schott.	*dwa ba, dwa g.yung*	tubers	eaten after being crushed and boiled	+++++
*Arisaema jacquemontii* Blume	*dwa ba, dwa g.yung*	tubers	eaten after being crushed and boiled	+++++
*Carum carvi* L.	*go snyod*	seeds	the crushed seeds are used as a spice	+++++
*Chenopodium album* L.	*sne’u*	leaves	stir-fried in oil after eliminating their bitter taste by boiling them long time in water, and are eaten with other food	++++
*Fragaria nubicola* Lindl. ex Lacaita	*‘bri ta sa ‘dzin*	fruits	children eat fresh fruits	++++
*Hippophae tibetana* Schlecht.	*to ra*, *star bu*	fruits	used to prepare a juice, fruits mainly eaten in the past	+++++
*Malva verticillata* L.	*lcam pa, bod lcam*	leaves	young leaves are eaten as vegetables, or stir-fried in oil before adding other vegetables and/or meat; leaves used to prepare a herbal tea	++++
*Polygonatum verticillatum* (L.) All.	*ra mnye*	leaves, roots	leaves eaten cooked, roots edible.	+++
*Polygonum vaccinifolia* Wallich ex Meisner	*ram bu*	roots, seeds	roots eaten fresh, flour obtained from ground seeds used as substitute for *tsampa* in the past	+++++
*Polygonum viviparum* L.	*ram bu*	roots, seeds	roots eaten fresh, flour obtained from ground seeds used as substitute for *tsampa* in the past	+++++
*Rhododendron anthopogon* D. Don	*ba lu, ba lu dkar po*	flowers and leaves	in the past used as substitute for tea	+++++
*Rosa macrophylla* Lindl.	*se ba*	fruits	fruits eaten fresh in the past, today by children	++++
*Rumex hastatus* D. Don	*sho mang, sha sna*	leaves	eaten as vegetables	++++
*Salvia hians* Royle ex Benth.	*‘jib rtsi, ‘jib rtsi sngon po*	stalks	stalks are eaten as vegetables	+++
*Stachys recta* L.	*bya pho rtse*	leaves	young leaves are eaten fresh as vegetables	+++
*Thymus linearis* Benth.	*smag tog pa*	leaves	mixed with chilly are used as condiment; used to prepare herbal tea	+++++
*Urtica dioica* L.	*zwa*	young shoots	used to prepare soups	+++++
**Plants collected in Dhorpatan, Baglung District, Nepal**				
*Arisaema jacquemontii* Blume	*dwa ba, dwa g.yung, kha tsha ba*	tubers	eaten after being crushed and boiled	++++
*Arisaema nepenthoides* (Wall.) Mart.	*dwa ba, dwa rgod, kha tsha ba*	tubers	eaten after being crushed and boiled	++++
*Arisaema utile* Hook. f. ex Schott	*dwa ba, dwa g.yung, kha tsha ba*	tubers	eaten after being crushed and boiled	++++
*Berberis angulosa* Wallich ex Hook. f. & Thoms.	*skyer pa, skyer nag*	fruits	eaten by children	+++
*Berberis aristata* DC.	*skyer pa, skyer dkar*	fruits	eaten by children	+++
*Capsella bursa-pastoris* (L.) Med.	*sog ka pa*	leaves	fresh leaves are fried with vegetables, dry leaves are eaten in local soups (*thug pa*)	++++
*Duchesnea indica* (Andr.) Focke	*‘bri ta sa ‘dzin*	fruits	fruits eaten fresh	+++
*Sinopodophyllum hexandrum* (Royle) T.S.Ying	*‘ol mo se*	fruits	children (maily in the past) eat fruits	++++
*Polygonum macrophyllum* D. Don var. *macrophyllum*	*spang ram, spang ram dmar po*	seeds, roots	roots eaten fresh, flour obtained from ground seeds used as substitute for *tsampa* in the past.	++++
*Rhododendron anthopogon* D. Don	*sur dkar, balu, ba lu dkar po*	flowers and leaves	in the past used as substitute for tea	+++++
*Rosa macrophylla* Lindl.	*se ba*	fruits	fruits eaten mainly in the past, today by children	++++
*Taraxacum officinale* G.H. Weber ex Wigger s.l.	*khur mang, ‘o ma me tog*	leaves	the leaves are fried in oil or cooked in water	++++
*Urtica dioica* L.	*zwa*	young shoots	young tender shoots are used to prepare a soup	+++++

*Consensus index. Indicates citation by % of informants. +: ≤10%; ++: 11-25%; +++: 26-50%; ++++: 51-75%; +++++: ≥76%.

**Table 2:** **The edible fungi collected in the study areas**

Fungi collected in Lithang County, Sichuan, China				
Botanical identification	Tibetan name	Part used	Use in local diet	C. index*
*Ophiocordyceps sinensis* (Berk.) G.H. Sung, J.M. Sung, Hywel-Jones & Spatafora	*dbyar rtswa dgun ‘bu, ‘bu*	entire fungus + larva	used to prepare an alcoholic beverage	+++++
*Tricholoma matsutake* (S. Ito & S.Imai) Singer	*be sha, be do shing sha mo*	entire fruit body	eaten without skin	+++++

*Consensus index. Indicates citation by % of informants. +: ≤10%; ++: 11-25%; +++: 26-50%; ++++: 51-75%; +++++: ≥76%.

**Table 3:** **Wild food plants according to The Four Tantras** [30] **and The Blue Beryl** [31]

Tibetan name	Botanical identification	Therapeutic properties
*ri sgog*	*Allium* spp*.*	All types of “mountain garlic” are heavy in quality and hard to digest, and increase appetite.
*lcum lo*	*Rheum palmatum* L.	Leaves from these two rhubarbs alleviate disorders arose from unbalance of the humour phlegm and improve appetite.
*chu lo*	*Rheum spiciforme* Royle	
*zwa*	*Urtica* spp*.*	Cooked nettles help to treat disorders arose from unbalance of the humour wind, generate heat, and aggravate bile and phlegm disorders.
*lcam pa*	*Malva verticillata* L.	When cooked, they generate heat and stop diarrhoea.
*de khur*	*Plantago depressa* Willd.	
*dwa ba*	*Arisaema* spp*.*	When cooked, it pacifies wind disorders, dries up abscesses, aggravates phlegm, and bile disorders.
*sne’u*	*Chenopodium album* L.	When cooked, it is harmful to eyes, and effective against constipation.
*mon sne’u dmar po*	*Amaranthus caudatus* L. *Chenopodium aristatum* L. (*Dysphania aristata* (Linnaeus) Mosyakin & Clemants) *C. botrys* L. (*Dysphania botrys* (Linnaeus) Mosyakin & Clemants)	When cooked, it pacifies all the three humours.
*khur mang*	*Taraxacum tibetanum* Handel-Mazzetti	When cooked, they are cool in quality, and alleviate hot disorders.
*skyabs*	*?*	
*sngo sga*	*Cremanthodium* spp.	When cooked, it cures hot disorders associated with bile, and relieves headache.
*lca ba*	*Angelica sinensis* (Oliver) Diels	When cooked, they relieve phlegm and wind disorders.
*ra mnye*	*Polygonatum cirrhifolium* (L.) All.	
*sgog sngon*	*Allium rubellum* auct. non Bieb.: Hook.f (A. *jacquemontii* Kunth) *Allium carolinianum* Redouté *Allium fistulosum* L.	When cooked, it cures hot and wind disorders.
***Spices***		
*g.yer ma*	*Zanthoxylum tibetanum* C*.* C. Huang. (*Zanthoxylum oxyphyllum* Edgeworth) *Zanthoxylum bungeanum* Maxim.	It opens the channels, but it increases phlegm and wind humours.
*go snyod*	*Carum carvi* L.	It cures poisoning and fever, promotes appetite and digestive heat.
*‘bam po*	*Ligusticum pteridophyllum* Franchet *Heracleum millefolium* Diels	It alleviates swellings.

**Table 4:** **Transliteration of the Tibetan terms mentioned in the article**

Phonetic transcription	Transliteration [29]
*aluk*	*a lug*
*arak*	*a rag*
*bam po*	*‘bam po*
*be khur*	*be khur*
*bu*	*‘bu*
*cha*	*ja*
*cha wa*	*lca ba*
*cham pa*	*lcam pa*
*che*	*dpyad*
*chiu kanlag*	*byi’u rkang lag*
*chölam*	*spyod lam*
*chon shi*	*cong zhi*
*chu lo*	*chu lo*
*chum lo*	*lcum lo*
*drebu*	*‘bras bu*
*drimo*	*‘bri mo*
*droma marku*	*gro ma mar khu*
*drönme shing*	*sgron me shing*
*duk*	*dug*
*dzo*	*mdzo*
*gö*	*rgod*
*go na shamo*	*sgo nga sha mo*
*go nyö*	*go snyod*
*kha tshawa*	*kha tsha ba*
*khawa*	*kha ba*
*khenta*	*khan da*
*khur mang*	*khur mang*
*kyur*	*skyur*
*laphuk*	*la phug*
*lusundün*	*lus zungs bdun*
*ma rikpa*	*ma rig pa*
*men*	*sman*
*mokmok*	*mog mog*
*mon neu marpo*	*mon sne’u dmar po*
*namshe*	*rnam shes*
*no ga*	*sngo sga*
*no ne*	*sngo ngad*
*no tshel*	*sngo tshal*
*nyalo*	*snya lo*
*nyepa sum*	*nyes pa gsum*
*pango*	*spang sgo*
*phap*	*phab*
*ramnye*	*ra mnye*
*ri gok*	*ri sgog*
*setshul*	*zas tshul*
*shabal*	*sha bal*
*shapakle*	*sha bag leb*
*shukdrum*	*shug ‘brum*
*solo*	*sro lo*
*tsa*	*rtswa*
*tsampa*	*tsam pa*
*tsha pö*	*tshwa spod*
*tsowase*	*‘tsho ba zas*
*ya*	*g.ya’*
*yalishing*	*g.ya’ li shing*
*yartsa gunbu*	*dbyar rtswa dgun ‘bu*
*yer ma*	*g.yer ma*
*yö char*	*g.yos sbyar*
*yung*	*g.yung*
*yung kar*	*g.yungs dkar*

The plant specimens gathered in the field were identified in collaboration with Professor J. F. Dobremez (Laboratoire d’Ecologie Alpine, Université de Savoie, France) and were deposited at the Herbarium of the Muséum National d’Histoire Naturelle de Paris, France. The specimens collected in Ladakh were identified and deposited at the Herbarium of the Botanical Survey of India, Dehradun, Uttar Pradesh, India.

### The study areas

**Figure 1:**
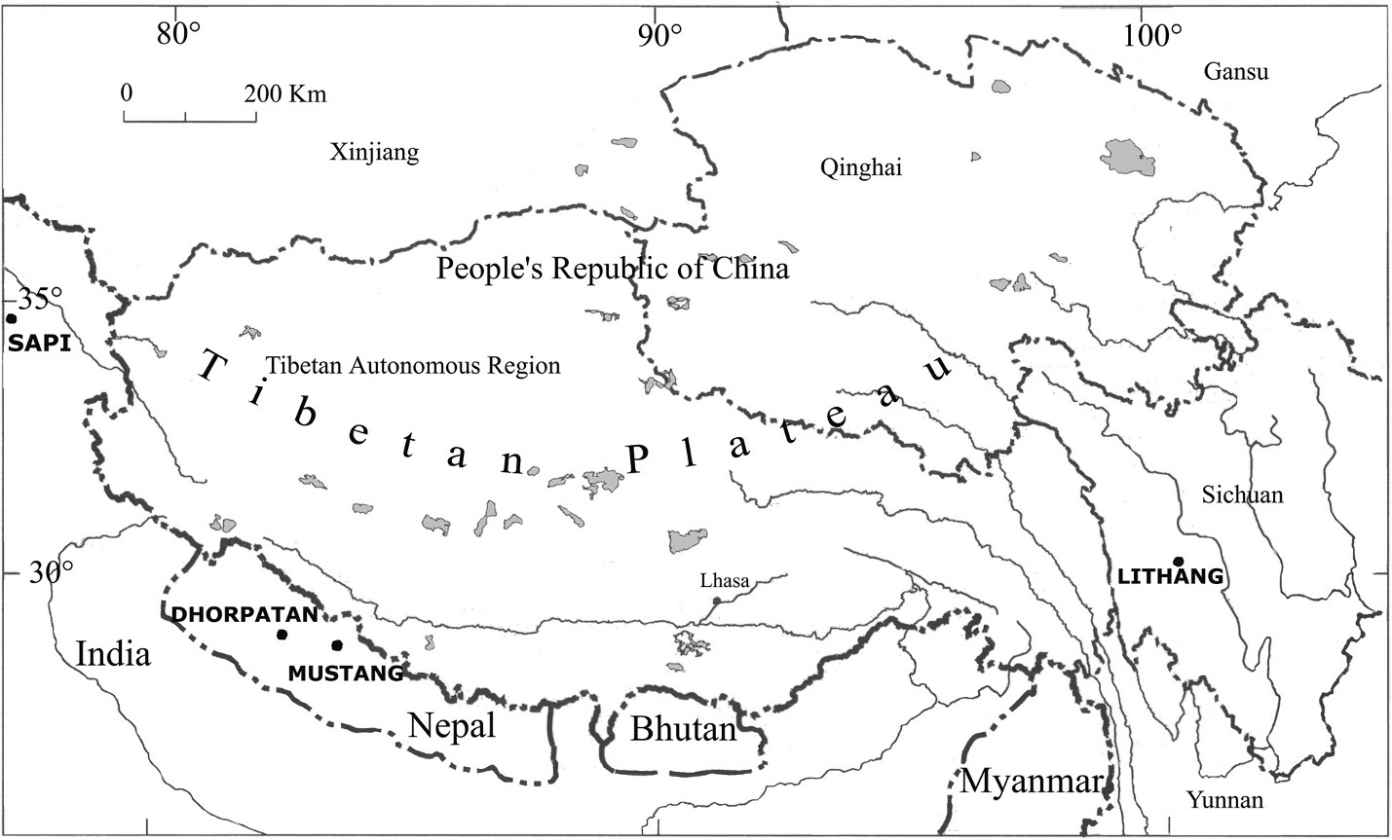
**Map showing the study areas.**

The study areas (Figure 1) represent some of the different environmental conditions that Tibetan people encounter throughout the vast area where they live. The eastern Tibetan plateau in western Sichuan Province (PRC), where Lithang township is located, the south Himalayan valleys in Dhorpatan (Nepal), and Khumbu, exhibit a climate, flora, and vegetation influenced by monsoon precipitations. On the contrary, the high valleys of lower Mustang District in Nepal, and Ladakh (Jammu and Kashmir, India), are not hit by the monsoon, and are dominated by aridity and cold.

#### Lithang County

Lithang County (North Latitude 29° 30’ - 30° 39’ – East longitude 99° 25’ - 101°) is located in the Ganzi Tibetan Autonomous Prefecture (Sichuan Province, China) over an area of 14,619 km^2^ [32]. The region is hit by the monsoon during summer, which brings the 80% of annual precipitations (500 – 1,100 mm.). Vegetation includes forests dominated by conifers as *Pinus densata*, *Abies squamata, Picea balfourina*, and *Juniperus* spp*.*, and oaks (*Quercus aquifolia*) [33]. Treeline is located around 4,200-4,400 m. Alpine zones host shrubs as *Rhododendron*, *Lonicera*, *Potentilla*, *Cotoneaster*, and *Caragana*, and herbaceous species including *Cyperaceae* (notably *Kobresia*), *Gramineae*, and many herbaceous plants as *Potentilla, Meconopsis, Gentiana, Aconitum, Delphinium, Anemone, Aster, Leontopodium, Anaphalis*, and *Thermopsis.* At higher altitude near the limit of vegetation *Saussurea*, *Saxifraga*, and *Arenaria* species are dominant [34]. Pasturelands occupy the 60% of the territory. The majority of the population is Tibetan (98%) [35]. Tibetan pastoralists live in the higher plateau areas whereas farmers and merchants live in the main towns, as Lithang the capital of the County, and in the lower forested valleys. Chinese immigration has been increasing in the past few decades.

#### Mustang

Lower Mustang is located in the southern part of Mustang District in central Nepal. The author spent most of his time at Jarkhot, a village located at 3,500 metres (28° 49’ North latitude and 83° 54’ East longitude). Local people speak a Tibetan dialect and are culturally Tibetan. Agriculture is mainly based on barley, grain, wheat, millet and potato cultivation. Dzo (a hybrid between the yak-bull and cow), sheep, and goats are reared. Dobremez [36] defined the climate of this region as “steppique”, very arid. Its vegetation consists of steppe formations, notably *Sophora moorcroftiana* and *Oxytropis sericopetala* steppe. Other species that manage to thrive in this extreme environment are *Berberis mucrifolia*, *Ephedra gerardiana*, *Lonicera hypoleuca*, and *Incarvillea arguta*. The next type of vegetation is the *Caragana gerardiana* and *Artemisia* steppe, which grows up to 4,200 m. It is richer in both herbaceous and shrub species (*Rosa sericea*, *Lonicera rupicola*, *L. minutifolia*, *Spiraea bella*, *S. arcuata*, *Ribes orientale*, *Berberis angulosa*). Towards the valley bottom, one may observe open xerophytic forests dominated by *Cupressus torulosa*. Higher up (4,200-5,100 m) *Caragana brevispina* and *Lonicera spinosa* steppe dominates the landscape. In the coolest places *Juniperus squamata*, *Potentilla fruticosa*, *Lonicera mirtillus* and herbaceous species may thrive. Over 5,000 m species with cushion forms predominate: *Potentilla biflora*, *Androsace sessilis*, *Thylacospermum rupifragum*, *Rhododendron nivale* and several *Saxifraga* [37].

#### Dhorpatan

The Nordzinling Tibetan settlement of Dhorpatan is located in Baglung District (Nepal), southwest of Dhaulagiri Himal. The region, which is included in the Dhorpatan Hunting Reserve, is situated in central Nepal 260 kilometres north west of Kathmandu. Dhorpatan (28°29’ North Latitude – 83° 05’ East Longitude) lies at an altitude of 2,850 metres in a valley extending from east to west at the southern border of the reserve. It can be reached in four days walking from Darbang after crossing the Jalja pass (3,400 m). In 1960 a Tibetan refugees’ settlement was established at Dhorpatan. Since then a fluctuating Tibetan population has been living there. According to Wilson [38], in 1981 there were 200-250 refugees in the area. The trend of the last decades showed a slow decrease in the number of the Tibetan residents. The settlement consists of five different camps spread along the valley. Being partially protected from monsoon winds, the region receives less precipitation (600-1,400 mm) than the other areas of the middle Himalaya. Xerophytic forests dominate between 2,800 and 3,000 m, in particular *Pinus wallichiana* forests which on south facing slopes are characterized by *facies* of *Juniperus indica*. At higher elevations (3,000-4,000 m) *Abies spectabilis*-*Betula utilis*-*Rhododendron campanulatum* forest is widespread. At around 4,000 m the alpine vegetation of the southern slopes of the Himalaya abruptly replaces the forests. It mainly consists of Gramineae and Cyperaceae, and artic and alpine genera as *Gentiana, Primula, Saxifraga, Pedicularis,* and *Polygonum*. The higher limit of the vegetation (4,900 m) is abrupt too [37].

#### Sapi

Ladakh (86,904 km^2^) is included in the Indian State of Jammu and Kashmir. Located between 32° 15 and 36° North latitude and between 75° 15 and 80° 15 East longitude, it is limited to the North-east and to the East by China, to the North-west by Pakistan, to the South and West by Himachal Pradesh and Jammu & Kashmir Indian States. A high plateau of 4,500 meters of mean altitude, Ladakh is located between Karakorum to the North and Himalaya to the South. The most important human settlements are spread along the main river valleys, particularly the Indus. Owing to extreme climatic conditions, population is scarce (around 260,000). Most people are culturally Tibetan and speak a Tibetan dialect. Small groups of Indo-European populations (Balti, Dards) live in Kargil and Leh Districts. Climate is very cold and arid (annual precipitation: 100-600 mm) [39]. It is mainly dominated by steppe vegetation (*Caragana*, *Artemisia*, *Stachys*, *Ephedra*, *Stipa*, *Acantholimum*) [40]. Mesophyte species are not widespread and hygrophyte scarcely represented. Few tree species manage to thrive as *Juniperus macropoda*, *J. indica* and *Betula utilis* in the most favourable spots, and also, as far as 5,000 meters, shrubs as *Myricaria, Lonicera, Caragana, Hippophae, Rosa, Berberis* and *Rhododendron* [39]. Sapi valley is located in the west part of Ladakh in Kargil District from 3,600 to 5,000 meters. Also inhabited by Muslim Balti, it is one of the most important areas of Ladakh for the collection of herbs. Owing to the greater amount of precipitations during winter, it hosts relatively lush vegetation including also alpine genera as *Leontopodium, Polygonum, Aconitum, Delphinium, Papaver, Rheum,* and *Ranunculus*.

## Results and discussion

In pre-modern Tibet the collection of wild food plants was most probably common practice. When investigating into this field one has to take into account that over the vast area traditionally inhabited by populations of Tibetan language and culture, geographical, climatic, floristic, vegetational, and cultural differences have influenced the types of plants exploited as food, their way of exploitation, and their significance in the local diet. For example the far-eastern and south-eastern Tibetan regions are hit by monsoon precipitations during summer, host lush vegetation, and have relatively high biodiversity. On the contrary in the cold and arid north-western Tibetan plateau plants manage to thrive only along rivers and streams, and at high altitude where water from melting glaciers and snow is available during the short summer. So Tibetans may live in regions that, as far as climate, flora, and vegetation are concerned, show contrasting features. That is why on high plateau areas the number of plants traditionally consumed as food is less abundant than in Tibetan south-eastern and eastern regions, and at the limit of the Tibetan plateau, where altitude decreases and biodiversity augments. This agrees with the author’s field data from Ladakh and with ethnobotanical research in Nepal Himalayan high valleys [11, 14, 15, 23, 24].

It nevertheless should be noted that the most important wild food plants having a significant use and role in the life of Tibetan people in both pre-modern and modern Tibet, according to the author’s knowledge, field experience, and available research data, grow, are used and/or are known in the majority of Tibetan regions.

Wild plants have not likely represented a crucial source of food for Tibetans, but have been mainly collected to be consumed as snacks during travels and summer transhumance, as spices, and to complete the local traditional diet often including few vegetables and fruits, particularly among pastoralists. Yet wild plants become important during famines, which have occurred at different times in the course of the centuries, the most recent ones that many informants from Lithang often evoke date back to the years of the Great Leap Forward (1958-1961) and of the Cultural Revolution (1966-1976).

At present grown vegetables and fruits are available in many Tibetan towns and villages and wild edible plants are collected only from time to time, except among pastoralists and possibly people from remote areas. The author observed Tibetans collecting wild food plants notably during the summer transhumance, and travels (on foot and by horse) between villages. Women from the study areas gather particular plants as the bulbs from some varieties of wild garlic (see below). Pastoralists of all ages and gender from Lithang County have good knowledge of and collect wild food plants, for example the well-known (among Tibetans) rhizomes from *Potentilla* (see below) to be consumed at their encampments and/or to be sold in Lithang market. It is worth noting that in the same county several young adults among the settled population do not have familiarity with wild plants whereas most adults and old people do. They remember plant names and use in local diet, and sometimes still gather and use them. So this knowledge may get lost forever in the near future, not being passed on to the new generations, who have changed their way of living and do not need any more to know about the natural environment and its exploitable resources. In Lithang Township, where the author spent most of his research time, several informants report that the use of wild food plants was more significant in the past, and particularly during the periods of economic hardship. At that time wild plants were collected to be consumed as vegetables (*no tshel*), fruits and seeds (*drebu*), and particularly to obtain substitutes for roasted barley flour (*tsampa*), and tea (*ja*), which in Tibet should be seen more as a foodstuff than as a beverage, being traditionally prepared by adding salt and butter. So nowadays in the study regions only a limited number of wild plants is used as food, more limited among settled population than among pastoralists and people living in remote areas. In the next paragraphs Tibetan knowledge and use concerning the most important plants consumed as food according to the author’s field data are examined.

Out of 75 total wild food plants and mushrooms listed in Tables 1 and 2, 32 specimens were collected in Lithang, 18 in Southern Mustang, 12 in Ladakh, and 13 in Dhorpatan. They belong to 36 genera and 60 species. 44 species are eaten as vegetables, 10 are used as spices\condiments, 15 as fruits (only children consumed 12 of them at the time of the fieldwork), 3 species are used as ferments to prepare yoghurt and beer, 5 as substitutes for *tsampa*, 4 as substitutes for tea, and 3 to prepare other beverages.

Data from Lithang, which are more representative, show that among the 30 wild food plant species locally exploited, 21 are consumed as vegetables, 5 as spices, 4 as fruits, 3 represent substitutes for *tsampa,* 2 substitutes for tea, and 1 is used as fermentation agent.

### Vegetables

Wild plants are consumed as vegetables, either fresh or dry, in which case they are usually conserved to be eaten during the long winter. Wild vegetables may be eaten in soups, boiled, stir-fried in oil with other vegetables and\or meat (see Table 1).

Informants report that some plants need a purification process before being consumed, for example the leaves of *Chenopodium* (*neu*) and the tubers of some species belonging to the Araceae family (*dawa*, *Arisaema* spp*.*). Well-known in Ladakh, lower Mustang, and Lithang, *Chenopodium* leaves are stir-fried in oil after eliminating their unpleasant taste (most informants used the word bitter, *khawa*, to indicate this) by boiling them long time in water, and are eaten with other food. Jest [11] informs us that people from Dolpo (north-west Nepal) believe that a poison (*duk*), named *ya* (“dirt”), covers *Chenopodium* leaves and that it is eliminated before use. Polhe [15] reports that in Nar (Manang District, Nepal) *Chenopodium* leaves are dried and eaten in winter as vegetables.

*Arisaema* species thrive in several Himalayan and Tibetan regions. Informants from Lithang, Southern Mustang, and Dhorpatan name these plants *dawa,* the designation also used in Tibetan medicine; others, mostly non-educated people from Lithang, report the name *kha tshawa*, “hot mouth”, since the taste of the tuber is hot. The most common way to classify this plant differentiates two types: *yung*, “domestic”, and *gö*, “wild”, the former grows at lower altitude often near villages and cultivated fields, the latter higher up in the mountains. Over Tibetan regions, each type may correspond to one or more botanical species (Table 1). This categorisation is also described in Tibetan medical treatises [41]. In Dhorpatan the author gathered two types of *dawa* (*Arisaema jacquenmontii* and *A. utile*) that local informants recognize. Overall, informants recognize for types of *dawa*, all of which thrive in the area. Traditional doctors named the two species that the author collected *dayung*, since they grow at relatively low altitude (2,800 – 3100 m.) in proximity to the settlement. One of the two “wild” types is most likely *A. nepenthoides* that the author collected at around 3,300 metres in the same area.

Informants from Lithang, Dhorpatan, and Southern Mustang report that in the past local communities used to consume the tubers of these plants only after eliminating their poison. They add that nowadays these products are rarely used and are only given to animals. Sacherer [14] reports that Sherpa people from Rolwaling valley in Nepal name this plant *aluk*, a designation that is evidently connected to the Nepali *alu*, which indicates potatoes. Sacherer observes that the term *aluk* could have been used in Nepal before the introduction of potatoes in the 19th century [42] to indicate edible underground organs rich in starch obtained from different plants. Sacherer adds that, if this is true, Sherpa people did not know this plant before their arrival in Nepal in the 16th century and at that time adopted the Nepali term indicating these products. Yet it is important to note that in eastern Tibetan regions, from where Sherpa people originate, several *Arisaema* species thrive, are locally well-known as the author’s research in eastern Tibetan regions has shown [6], and were most likely known also in the past. Also, *dawa*, as mentioned above, is used in Tibetan medicine and it is described in several traditional treatises. Among them it is worth mentioning a Tibetan *materia medica* treatise [41] compiled in eastern Tibetan regions, which dates back to the first half of the 18th century. Its author mentions the term *aluk* as a synonym for *dawa.* So the term *aluk* was possibly used over a larger area.

Informants from Dhorpatan, lower Mustang, and Lithang report that *dawa* underground organs need to be crushed and boiled for long time before eating. This procedure is meant to eliminate the poison present in the tuber. Hooker [12] reports that in northern Sikkim, at the junction of the Zemu and Thlonk rivers, near plentiful *Arisaema* plants, local Tibetan people had dug several small holes in the soil, in which wood poles had been introduced. They were used to crush *dawa* underground organs consumed as foodstuff during spring by local populations. After being crushed, the tubers were left in the holes filled with water. After a week they started to ferment, a signal that the poison had been eliminated. At this time the tuber, transformed into a fibrous acid lump, could be boiled and eaten. Yet Hooker adds that the consuming of this product often entailed digestive problems, skin and hair loss, notably when fermentation was not sufficient to dissipate toxins. According to Polunin and Stainton [43] “The tuberous roots of many [*Arisaema*] species can be ground into a flour and eaten. Care must be taken as the tubers contain minute sharp particles, which can damage the digestive tract”.

Tibetan people know *sa,* “nettles” (*Urtica* spp.), very well; this is the food of hermits, and Tibetans traditionally consume it as foodstuff. In Ladakh (in Sapi, Kortshog and Kanji villages, *Urtica hyperborea*), in Lithang (*U. triangularis*), and in Dhorpatan (*U. dioica*), the author observed villagers and pastoralists collecting this plant, the young leaves of which are used to prepare excellent soups. Otherwise the plant is dried and stored to be consumed during winter. Jest [11] reports that in May women from Dolpo (north-west Nepal) collect nettle (*U. dioica; U. hyperborea*) stalks and leaves and keep them in baskets. They are used to prepare soups and sauces.

The well-known *droma* (*Potentilla anserina*) was frequently used as foodstuff in pre-modern Tibet [44]. Today its rhizomes are consumed occasionally, for example by pastoralists from Lithang County and most likely from other areas. At the time of the fieldwork in Lithang market pastoralists sold 500 grams of *droma* rhizomes for 5 yuan (50 cent euro). In Lithang *droma* rhizomes were and are collected mainly in autumn when, according to informants, their taste is sweeter and the size is bigger. They are boiled in water until they become soft, the volume of the water has to be two times the volume of the rhizomes and, having taken a sweet taste at the end of the cooking, may be drunk. Melted butter is poured on the cooked rhizomes, the mixing is cooked again for a few minutes, and *droma marku*, “melted butter with *Potentilla* rhizomes”, is ready. This food may also be consumed with yoghurt and/or parched barley flour. Traditionally this plant is included among the courses prepared during festivals. According to Rinzin Thargyal [45] in pre-modern Dege Kingdom (in today north western Sichuan Chinese Province) women used to dig *droma* in spring and autumn, “its harvest itself could not supplement the household income, but it was indispensable for the New Year celebrations during which a dish called *droma marku* was eaten.” Similarly Jest [46] reports that in Dolpo (Nepal) this plant was included in a recipe composed of nine ingredients, prepared for New Year celebrations. It included barley, wheat, rice, cheese, roots of *Potentilla*, bamboo shoots, peas, mutton, and salt. Also Duncan [8] writes that at Bathang (in today western Sichuan Chinese Province) “*Droma* are the conventional present in the spring at festival season; and are important at wedding as the first food given to the bride when she arrives at the groom’s home upon the betrothal night”.

*Rambu* (*Polygonum* spp*.*) has been collected as a plant food in many Tibetan regions, and notably has represented an important source of food during famines. Informants from Lithang, Dhorpatan, and southern Mustang report that its roots can be eaten fresh and that its ground seeds were consumed in the past mixed with *tsampa.* Some of them state that the *rambu* roots that are internally reddish in colour are very sweet and the most sought after, as the ones from the plant specimen collected in Dhorpatan (*Polygonum macrophyllum* var. *macrophyllum*). Rockhill [9], who visited eastern and north-eastern Tibetan regions, reports that: “On the sides of the mountains overlooking the Rama ch’u, we passed a number of women picking ramba, the seeds of which, when dried and ground, are mixed with tsampa and eaten, and this adds one more to the very small list of native dishes. These women told me that it was jimbo, jimbo ré, “very, very good”, but they are not hard to please”. Polhe [15] informs us that *rambu* “was formerly an important nutritive plant for the Manangis, serving to supplement the harvest of grain. The spike-like flower clusters were gleaned in September/October, the nutlets dried, ground and roasted like *tsampa*. From the unroasted flour flat round breads were made and notably that since today [1984-1985] there is no longer any dependence on a supplement to the grain harvest, the spikes are no longer gleaned.” Sacherer [14] reports that in remote areas as Rolwaling valley in Nepal in the 1970s local Sherpa people still consumed as snack during the summer transhumance the underground organs of *monzo* (*Bistorta macrophylla,* synonym for *Polygonum macrophyllum* var. *macrophyllum*, which corresponds to *rambu* in Dhorpatan) and *tai monzo* (*Bistorta* sp.). These two denominations are most likely local synonyms for *rambu*.

A few wild plants are regularly consumed as vegetables by travellers and herders. People from Lithang collect and eat on the spot, after removing the skin, the stem of two kinds of rhubarb, *chukyur* (*Rheum alexandrae*) and *chum* (*Rheum palmatum*), and of Himalayan Knotweed, another polygonaceae named *nyalo* (*Polygonum polystachyum*). *Chukyur* is so named because the abundant water (c*hu*) that its stem contains is sour (*kyur*) in taste. Similarly herders from Ladakh often consume petioles and stems of another rhubarb named *chutsa* (*Rheum spiciforme*). Informants from Leh (Ladakh) mention a wild plant designated *sat*, also mentioned by Rizvi [47], which merchants travelling from Ladakh to west Tibet used to gather and eat. Rizvi informs us that a mixture with *tsampa* was prepared, and that this plant was the only food consumed in eastern Ladakh regions during 10 days trip. The term *sat* might correspond to the Tibetan name *srad*, pronounced *sat* in Ladakhi Tibetan dialect. It is generally used to indicate wild Fabaceae, among them several species belonging to *Hedysarum* and *Thermopsis* genera.

### Fruits

It is interesting to note that the wild fruits regularly consumed in pre-modern Tibet according to travellers’ accounts [9] are nowadays almost only eaten by children from the study areas. For example in Lithang *ölmose* (*Sinopodophyllum hexandrum*) fruits, in Southern Mustang, Khumbu, and Ladakh *tarbu* fruits (*Hippophae tibetana, H. salicifolia, H. rhamnoides* subsp. *turkestanica*), in Southern Mustang, Dhorpatan (and Khumbu) *drita sadzin* (*Fragaria nubicola, Duchesnea indica*) and *sewa* (*Rosa macrophylla*) fruits, in Dhorpatan (*Berberis aristata*, *B. angulosa*) and Khumbu (*Berberis* sp.) *kyerpa* fruits.

### Spices

Tibetan people from the study areas know a few plants that may be used as spices in local cuisine recipes. The majority of them are well-known over Tibetan regions. *Gokpa*, wild garlic (*Allium* spp.), of which Tibetans recognize several types having different flavour and qualities, is by far the most important plant traditionally used as spice (and vegetable). Many informants from different regions report that its bulbs also help to relieve high altitude headache. In Lithang informants recognize three or four types of it: *chiugok*, “small bird’s garlic” (*Allium macranthum*)*, rukgok* (*Allium prattii*), *shagok*, “meat? garlic” (*Allium* sp.), and *gokpa* (*Allium* sp.). Also in southern Mustang informants distinguish a few types of this plant. The most significant distinction separates the varieties named *gokpa* from *dzimbu* (*Allium roseum*). The latter is deemed to be more valuable than the others since, as also Sacherer [14], who worked in Rolwaling valley (Dolakha District, Nepal), reports, it is the only one that can be well conserved throughout the entire winter. People from Lithang prepare a sauce with garlic. The entire plant is cleaned and cut into small pieces. It is mixed with butter, tea, roasted barley flour (*tsampa*), salt, and chilly. *Tsampa* may not be added. The sauce so obtained is eaten with Tibetan dumplings (*mokmok*) and fried meat-filled pancakes (*shapakle*).

Informants from southern Mustang and Sapi (Ladakh) report that *Thymus linearis,* respectively named *maktokpa* (by local traditional doctors) in the former and *sulu* in the latter region, leaves and stems mixed with chilly are used as condiment. The same use is reported by Polhe [15] concerning Nar region (Manang District, Nepal). According to Jest in the 1960s in Dolpo (Nepal) *Thymus serpyllum* [*T. linearis*]^a^ (locally named *gothok marcha*) leaves were also used as a spice, after having been crushed with salt and *laphuk* (*Raphanus sativus*) roots, and mixed with curdled milk, tea, or water. The result was a condiment used to flavour food, for example *tsampa* dough.

### Beverages

Tea is by far one of the most important products in Tibetan traditional diet and economy. That is why Tibetan people selected from the wild several substitutes for it, which were used when tea was lacking, an event that was not rare in pre-modern Tibet. Several informants from Lithang recall a plant, named *tönja*, “autumn tea” (*Potentilla* sp.), that was used to prepare tea when the real one did not arrive from China. The aerial organs of the plant were dried in the sun, and then boiled in water for long time. The scented flowers and leaves of *surkar* (*Rhododendron* sp.) were used as substitute for tea in Lithang, Southern Mustang, and Shar Khumbu (in the last two regions it is named *balu karpo* and its botanical identification corresponds to *Rhododendron anthopogon*), and also by poor people in remote Dolpo, where tea was seen as “a beverage of rich households” [11]. Tibetans from Dhorpatan give the same information. Some of educated informants from Lithang report that nowadays *surkar* tea is sometimes used by traditional doctors for relieving blood diseases. Concerning pre-modern Tibet Rockhill [10] explains that in eastern Tibet willow (*Salix* sp.) leaves were used as substitute for tea and that, when tea was lacking, any plant substance that could give some colour and flavour to water was sought for. Bell [48] likewise informs us that poor Tibetan people who could not afford to buy tea, used some plant substitutes as maple leaves (*yalishing, Acer caudatum*) in Sikkim. The same utilisation of maple leaves was also common in Shar Khumbu, as the author’s informants report. Sacherer [14] reports that in 1977 in Rolwaling valley *pemakoko* (*Epilobium conspermum*) dried leaves were used as a substitute for or were added to tea, because the latter was too expensive, being imported in the region on foot, a travel that took eight days.

Seabuckthorn (*Hippophae* spp*.*) represents a well-known plant in the Tibetan world. It has been used in local diet, popular and learned Tibetan medicine, and nowadays it is also commercially exploited by locally established companies. People from Jarkhot and adjacent villages in southern Mustang prepare a juice from the fruits of this shrub (*tora*, *Hippophae tibetana*). Today it is drunk with sugar, in the past without. Informants report that a concentrate (well-known among Tibetan traditional doctors as *khenta*) of the juice is prepared through boiling the fruits for long time and it is added to pickles and several food recipes. In Ladakh children used to roll a big leaf up to form a cone used as a container. The juice obtained by squeezing seabuckthorn (named *tshogkyur* in Sapi, *Hippophae rhamnoides* subs. *turkestanica*) fruits was collected in the cone and drunk. At present a local company based in Leh (Ladakh Foods Ltd in a joint venture with the Small Farmers’ Agri-Business Consortium and the National Agricultural Cooperative Marketing Federation of India Ltd) produces and sells on the market jam and juice from seabuckthorn fruits [49]. Also, in 2010 the Union Environment and Forest Ministry and the Defence Research and Development Organisation launched a major initiative for seabuckthorn cultivation in the Himalayas. Women’s self-help groups in Jammu and Kashmir (and Ladakh) will be included for the project, which is expected to provide employment opportunities [50]. Informants from Lithang state that seabuckthorn fruits (possibly obtained from the species *Hippophae litangensis* [51], a specimen of this plant has not been collected by the author during his fieldwork in Lithang) growing in the region are nowadays much less consumed than in the past before the 1950s.

In Lithang the few people who consume the well-known *yartsa gunbu* (*Ophiocordyceps sinensis*, a parasitic mushroom that attacks the larvae of *Thitarodes* moth species) typically do so in the form of a beverage that is prepared at home in different ways. This drink is not only consumed as a tonic and aphrodisiac, but also as hard liquor. The author observed Tibetans sharing these beverages kept in tiny bottles while conducting activities such as carving religious prayers on stones, gambling, and taking picnics in the summer pastures. Informants explain that these drinks are prepared by dipping a few *yartsa gunbu* specimens into a container filled with *arak*, a local alcoholic spirit processed from barley or rice. The number of specimens may vary according to the quantity of *arak* held in the container and the strength required. Usually 3 to 5 specimens of *yartsa gunbu* are used for each half-litre of *arak*. The drink is ready after having been kept in a cool place for 2-3 months. Some people wait a year or more before consuming it, claiming that the long period of the drug permanence in the alcohol increases the beverage flavour and potency. It is worth mentioning that Tibetans traditionally conceive *yartsa gunbu* as a plant, and particularly as a “grass”. It is seen as a single substance or phenomenon, which is subject to a metamorphosis occurring from the beginning of spring to the early summer. The Tibetan designation of that substance is very accurate and shows a good knowledge of its biological and seasonal changes based on exact observations in the field. Indeed, this appellation, which is a compound word formed by four nouns meaning “summer-grass winter-worm”, gives all the necessary explanations to understand its cycle and transformation. Tibetans believe that during winter the *yartsa gunbu* lives as a worm (*bu*) and that, after a metamorphosis occurring at the beginning of spring, it changes into a kind of grass (*tsa*) [19]. According to Tibetan people [6], the category designated *tsa* includes all the various common wild plants with narrow green leaves that are of little dimension and flexible nature, that are fixed to the ground by means of underground structures, and that are eaten by yaks and sheep. Actually, the fruiting body of the fungus is very similar to a blade of grass as concerns size and general aspect.

### Wild food plants use in case of famine

“Food substitution is the most common individual subsistence strategy in times of want and starvation. Indeed, all the early studies on the use of wild food plants in Europe – from those coming from the 19th century until more or less the 1960s – capture the memory of famine and the use of wild plants as a means of basic survival, including the consumption of starvation foods that in normal times would be discarded by the community” [52]. This assertion is also valid for Tibetan cultural regions. According to the author’s field data, which are homogeneous throughout the study areas, two kinds of poverty food are prominent in people’s memories: plants to produce substitute flours and plants used as vegetables and to make soups, the former ones being more important than the latter ones since the staple diet of Tibetan populations is based on roasted barley flour (*tsampa*).

In case of famine, *bedo shing* fruits (*Quercus* sp.), *rambu* (*Polygonum viviparum*) underground organs and seeds, and *droma* (*Potentilla anserina*) rhizomes were usually collected in Lithang. The first two plants represent the most important natural products used as substitutes for *tsampa*. Informants from Lithang report that oak acorns were boiled for long time in water, dried, and ground. Oak acorns are to be seen as a starvation food that in normal time local people would discard. Informants from the study areas (except Ladakh) informs us that *ram bu* rhizomes were eaten fresh after eliminating the skin, or rhizomes and seeds were dried and ground. They add that at present only pastoralists sometimes produce flour from this plant, which is given to yaks, goats, and sheep. Jest [11] reports the same use of *ram bu* seeds during famines in Dolpo (north-west Nepal), which were lightly parched, then powdered, and eaten as *tsampa* or mixed with curdled milk. Informants from the study regions report that the flours obtained from different plants were often mixed.

The third important poverty food was *droma.* Its rhizomes were still consumed at the time of the fieldwork in Lithang. Stein [44] comments that in Tibetan epic tales it is frequently mentioned that hunting marmots, consuming their meat and *droma* rhizomes found in their burrows, means to live away from one’s own country because forced to do so.

Informants from Lithang affirm that during famines nettles (*Urtica triangularis*) were also important sources of food, particularly sought for at the beginning of spring. Sacherer [14] reports that in autumn 1972 when a famine struck the entirety of Nepal, Sherpa people from Rolwaling collected different kinds of *Arisaema* (*dawa*) rhizomes that, as it has been mentioned above, were regularly used as foodstuff in the past.

### Mushrooms

Pastoralists and settled people from Lithang regularly collect, eat, and trade mushrooms, and particularly the ones growing in the lower forested valleys. This may also be affirmed referring to people living in eastern Tibetan regions, which benefit from similar climatic and vegetational conditions [18]. A few explorers and missionaries that travelled and lived in pre-modern Tibet substantiate this. For example according to Rockhill [9] mushrooms possibly represented a significant resource to several Tibetan communities from eastern Tibetan regions. He reports that Tibetan people from Chamdo ate a mushroom designated *shara*, whose cap is yellow above and white below, and informs us that “fried with butter, they tasted like crêpes à la Bordelaise”. According to the missionary Duncan [8], people from Bathang region used to collect mushrooms, cut them into pieces, which were dried strung with threads under roof cornices. They were usually eaten with soups.

The author could not identify most of the mushrooms that he collected in Lithang, yet it is interesting to present here the use of some of them. *Besha* (*Tricholoma matsutake*) is by far the most important mushroom (after *Ophiocordyceps sinensis*) collected today by local people, trade in which plays an essential role in present economy. The fungus is sold to Chinese merchants and mainly exported to Japan [18]. Informants from Lithang County state that *besha* (*bedoshing shamo*), “oak mushroom”, connotes a fungus (*shamo*) that mainly grows in oak (*bedo*, *Quercus* sp.) and pine (*drönme shing*) forests in the lower valleys (*rong*) of the County. The fungus is collected mainly in August. It is worth noting that the popular Tibetan designation *besha* appropriately indicates *Tricholoma* symbiotic partnership with oaks. Local people fried fresh or dry *besha* in oil. The drying process is made by hanging the fungi on the ceiling, threaded with a string. These mushrooms are always cooked without skin and consumed with vegetables. Their price is relatively high (200 Yuan - 25 USD at the time of the fieldwork - for 500 grams of the best quality) and few people can afford to buy them. Traders describe the best *besha* specimens as follows: the stem is 4 fingers long, the cap should not be wide-open, its organs must be hard when touched, it must not be black, and it must not host worms. In the same area, the cap of a mushroom named *pango* (“meadow egg”, non-identified specimen) is heated in the ashes. When hot, it is used as container to stuff other food items, notably salt, *tsampa*, and butter. It is then eaten. Another mushroom consumed in Lithang is *chiukanlag*, (“small bird’s legs”, non-identified specimen), so named because its fruiting body has several ramifications that are very similar to small bird’s legs.

It is worth noting that educated and non-educated informants from the study areas often talk about poisonous mushrooms, which must be avoided. According to many of them the sun’s rays contain a kind of poison, and sun potency is so strong that it can negatively influence plant and mushroom qualities. So when, after completing their growth, mushrooms remain in direct sunlight for a few weeks (two weeks according to many informants), they absorb the poison contained in sun rays and become poisonous. Another sign that allows people to determine mushroom poisonousness is the presence of worms. Some informants (both educated and non-educated) from Lithang say that concerning certain mushrooms as *go na shamo* (“egg mushroom”, specimen not-identified) one has to be careful not to touch it with iron tools: “if (the mushroom) touches iron, it will contain poison.” Yet they do not provide any explanation to this phenomenon.

### Plants as starters of fermentation processes

In Tibetan traditional societies ferments are mainly used in the preparation of yoghurt from *drimo* (yak female) milk, and of beer from barley. Informants from Sapi (Ladakh) describe the way to prepare *phap*, the agent starting the fermentation process. They report that powdered leaves and flowers of *chagöpö* (*Delphinium brunonianum)* and *burtse* (*Artemisia gmelinii* var. *gmelinii*) are kneaded with wheat flour and water. The paste so obtained is moulded into small ball-shaped tablets. They are kept in a dark room wrapped in a woollen cloth for 15-20 days, and then dried. According to Sacherer [14] in Rolwaling valley (Nepal) two plants are collected and used as yeast in the preparation of beer: *kemba girbu* (*Artemisia vulgaris*) and *kemba tikpe* (*Waldheimia glabra*). Millet flour balls are wrapped in the leaves of the two plants. After a few days their surface becomes whitish owing to the development of mushroom hyphae. At this stage the leaves are removed and the balls, now named *phap*, are placed in a basket and fumigated. Similar processes are possible employed in other Tibetan regions as Meyer [28] reports concerning the Nepalese Dolpo region.

In Lithang at the time of the fieldwork ferment tablets were sold by Chinese traders in the food market, so possibly yeast is not any more locally prepared as often as in the past. Yet several informants report that stalks and leaves of *santsikarpo* (*Galium aparine*), after being thoroughly rubbed between hands, are added to milk to make yoghurt.

### Hermits’ wild food plants

Almost all informants report that in both present and past times hermits have managed to live and meditate for many years in mountain caves by consuming as food just few wild plants collected in the area surrounding their retreats. Legends and histories concerning the life of Milarepa (1051-1135), the most famous hermit sage from Tibet [53], of other hermits as the autobiography of Godrakpa (1170-1249) [54], and people from the study areas, offer some information on the plants eaten during the long seclusion.

From nettles a substitute of dull flour may be obtained, and nutritious soups are prepared. Nettles (*Urtica hyperborea*, *U. dioica, U. triangularis*) are common over Tibetan regions where they can thrive up to 5,000 metres and are locally used as foodstuff (see above).

Godrakpa, a teacher and yogin who rigorously practised meditation, consumed few wild products while living in isolated mountain ranges: *shabal* (mosses and lichens), *solo* (*Rhodiola* spp*.*), and *nyalo* (*Polygonum polystachyum*) [54]. Informants from Lithang report that *rambu* (*Polygonum viviparum*) seeds, leaves, and roots, and *droma* rhizomes are also eaten by hermits, and that Milarepa used to eat the basal leaves of *ripa*, a non-identified herbaceous plant having a particular fragrance. A Tibetan doctor from Jarkhot in southern Mustang District reports that a Lama from his same Buddhist tradition spent several years in retreat nourishing himself only with juniper seed cones (*shukdrum*). Yet according to several informants from the same area this plant’s fruit is not edible owing to its strong sour taste.

### Wild food plants in Tibetan medicine

Tibetan medical theory is rooted in the naturalistic Eurasian medical traditions (Greek-Persian, Indian, and Chinese) and in “a series of philosophical presuppositions shared by all Buddhist traditions concerning the nature of the phenomenal world, the fabric of the material environment, the physical components and psychic factors which constitute the beings inhabiting it.” [55] These presuppositions include the elemental theory of matter, the notion of the mind, the law of karma, and the concept of the existence of subtle channels pervading the human body, notions integrated from Indian philosophic and religious traditions, tantrism, and yoga. Between the 7^th^ and the 12^th^ century this heterogeneous knowledge has been integrated, and a homogeneous and rational theory, which makes Tibetan medicine independent from the traditions that influenced it, was devised.

The body is essentially seen as a microcosm of the natural environment, which is the macrocosm. Biological matter is conceived as directly originating from mental defilements, particularly ignorance (*ma rikpa*) that produces ego sensation, which gives rise to the “three (mental) defects” (*nyepa sum,* delusion, anger, and attachment). These defects keep human beings bound to *samsara*, the continual repetitive cycle of birth and death. At the moment of conception, the conscious principle (*namshe*), pushed to reincarnate by the “three defects” that are present in it, combines with male and female reproductive fluids. During the embryo development it is these very three defects that give rise to the three humours (wind, bile, and phlegm), bodily fluids responsible for all its functions, both physical and psychological. The three humours play a significant role in the digestive process and in the metabolic chain engendering from it. Ingested food, and notably chyle produced by digestion, which is defined as the first of the “bodily seven constituents” (*lusundün*)*,* is the material from which the other six constituents (blood, muscles, fat, bones, marrow, and reproductive fluids) are produced through a metabolic chain. So eating habits affect the way constituents are formed, and their health, and particularly reproductive fluids produced at the last stage of the chain. That is why Tibetan medical practitioners emphasize that diet and digestion are so important and see diet as a way of both curing diseases and maintaining a healthy body. Particularly dietetics (*setshul*) represents the second method of therapy in order of strength after mode of conduct (*chölam*), before medicaments (*men*), and external treatments (*che*).

Wild food plants, as grown food plants, are also seen as medicinal substances having their own qualities and potencies, which originate from their elementary composition and from other criteria related to plant place of growth, intrinsic nature, smell, and the theory of signatures [6]. So wild food plants, included in the category named “life sustaining diets” (*tsowase*), can be used to counteract different types of diseases. Yet their action is milder than the one of the substances included in the categories of “medicine” (*men*).

The Four Tantras, the fundamental text of Tibetan medicine, probably composed in the 11^th^ -12^th^ century, [30], and its well-known commentary the Blue Beryl (*Vaidurya sngon po,* 17th century) [31], both of which are still today crucial reference treatises for Tibetan medical students and practitioners, categorize food according to its types and qualities (Explanatory tantra, Chapters 16 and 17). Also, food substances are illustrated in a series of medical thangkas (paintings on canvas) that the Regent Sangye Gyatso commissioned in the second half of the 17^th^ century [56]. This work is the illustrated commentary to the Blue Beryl. Among the 79 paintings devoted to representing all specialisations of Tibetan medicine, the 21^st^ and 22^nd^ of the series illustrate the foods described in Chapters 16 and 17. Among the many animals and plants described and illustrated as food, some wild food plants that nowadays Tibetans still collect and use in local diet are included (Table 3), of which the majority have already been mentioned above. Tibetan people certainly collected and consumed these wild plants at the time of the compilation of these treatises and before it. The section including aromatic herbs and vegetables (*no ne*) describes *ri gok*, “mountain garlic” (*Allium* spp.)^b^, and the leaves of two rhubarbs: *chum lo* (*Rheum palmatum* leaves) and *chu lo* (*Rheum spiciforme* leaves). The section listing cooked food (*yö char*) includes *sa*, (*Urtica* spp*.*), *cham pa* (*Malva verticillata*), *be khur* (*Plantago depressa*), *dawa* (*Arisaema* spp*.*), *sne’u* (*Chenopodium album*), *mon*^*c*^*neu marpo* (*Amaranthus caudatu*s^*d*^ [57] *, Chenopodium aristatum*^*e*^ [56], *C. botrys*^*e*^ [56]), *khur mang* (*Taraxacum tibetanum*), another type of dandelion (*kyap*)^g^ [41], *no ga* (*Cremanthodium* spp*.*), *cha wa* (*Angelica sinensis* [57]), *ramnye* (*Polygonatum cirrhifolium* [57, 58]), *gok nön* (*Allium rubellum*^*h*^ [56] *, A. fistulosum*, and *A. carolinianum*^*i*^), and *yer ma* (*Zanthoxylum tibetanum*^*j*^ [56] *, Zanthoxylum bungeanum* [57, 58]) in the section devoted to representing salts and spices (*tsha pö*). Two more items are illustrated in the medical thangkas as an example of mixed spices, but not in the Four Tantras and Blue Beryl: *go nyö* (*Carum carvi*), and *bam po* (*Ligusticum pteridophyllum* [57] *, Heracleum millefolium* [58]). In chapter 17, devoted to dietary restrictions (concerning poisoning and incompatibility), mushrooms (*sha mo*) and again dandelion are mentioned. The former must not be fried in mustard (*yung kar*) oil. Calcitum powder (*chon shi*), dandelion, and mushrooms are incompatible food.

## Conclusions

The data obtained in the study regions show that Tibetan people traditionally exploit a limited number of wild food plants. These have possibly never represented crucial food items, but could mostly help reduce the lack in vegetables and fruits in traditional Tibetan diet, and particularly among pastoralists. Wild food plants are far more important during famines, being mainly used as substitutes for the staple Tibetan food: roasted barley flour (*tsampa*).

Today only a few among the traditionally used wild food plants are regularly collected and consumed by Tibetan people, less in the main towns and villages, moreso in remote areas and among pastoralists.

A limited number of wild edible plants are well-known and/or exploited over Tibetan regions as the different types of *gokpa* (*Allium* spp*.*), *droma* (*Potentilla anserina*), and *rambu* (*Polygonum* spp*.*). The author could verify this during field study and many journeys to other Tibetan cultural regions. Reference studies and information on traditional texts also agree with the author’s findings.

Some food plants are collected only occasionally, others are mainly known by old people, and are rarely or not used any more. Younger generations from Lithang town, and most probably from other towns in Chinese Tibet and other Tibetan cultural regions, where the way of living has significantly changed in the past few decades, have completely lost this knowledge. This mainly happens where modernisation and social transformations are more important and grown vegetables and fruits are available in local markets.

Data obtained in Dhorpatan are interesting since they show that Tibetan people established there since 1960 have started to select the same wild food plants that they used to collect and consume in their home villages in Tibet. This process was conducted by taking as ideal models the edible plants from their home regions, so by using morphological, biological, and other features that can be perceived by sense organs. Table 1 shows that, as far as their botanical genus is concerned, all the edible plants from this area nearly correspond to the ones exploited in the other Tibetan regions.

Unfortunately in-depth studies in this field are rare, particularly concerning Tibetan regions belonging to China.

It is interesting to note that several studies, for example the ones conducted in Heihe and Dali Valley in Qinling Mountains (Shaanxi, central China) [59, 60], have shown that Han people from the regions located to the east of ethnic Tibet, as well as some Tibetan communities in lower elevations on the eastern edges of the Tibetan Plateau [21, 22] use a large number of wild vegetables as food plants.

Tibetan peoples living in high plateau areas of the Tibetan Autonomous Region, parts of Qinghai, Gansu, Sichuan, and Yunnan Provinces, have developed their own peculiar knowledge and traditions concerning the concept and use of the natural world, which are distinct from the Chinese ones. Concerning food-plants, only a limited number of greens are locally available. Growing as “greens” in the severe climatic conditions of Tibet does not usually represent an advantage, and plants species had to devise particular adaptations to survive. Only from the 1950s onwards Tibetans have come into stable contact with the Chinese when they took over these regions. Before that time, travelling to Tibet was not easy due to its extreme remoteness, harsh weather, and difficult geography. On the contrary at the periphery of the plateau Tibetans have lived in contact with other people and sometimes have been influenced by them, also in wild food-plant use. This has been shown [21] among the Tibetans from Diqing Tibetan Autonomous Prefecture in northwest Yunnan: their knowledge of wild food-plants seems to have been influenced by Lisu, Naxi, Han Chinese, and other ethnic groups living in the area.

Today many Tibetan regions have been opened up to modernisation and globalisation processes and are influenced by national and international economic transformations and trends. So local people specialise in collecting natural products that are increasingly demanded in both China and abroad. Among them there are several medicinal and food substances as notably mushrooms as caterpillar fungus and matsutake. At present Tibetan people strongly benefit from these activities.

## Endnotes

^a^According to the Annotated Checklist of the Flowering Plants of Nepal [61] the only species belonging to the genus *Thymus* is *T. linearis*.

^b^Concerning the botanical identification of the plants that the author has not mentioned before and that he did not collect and identified, the ones available in modern Tibetan *materia medica* and medical treatises are proposed. One should understand that variability of plant identification is constant throughout the vast area where Tibetans live. Thus other botanical identifications may occur in other Tibetan regions.

^c^The Tibetan term *mon* indicates those regions that are not any more inhabited by Tibetan peoples, located to the southern and south-eastern fringe of the Tibetan plateau.

^d^This species is widely cultivated. in China. In a traditional treatise it is stated that this plant grows in house gardens [41].

^e^The binomial *Chenopodium aristatum* is the basionym of *Dysphania aristata* (Linnaeus) Mosyakin & Clemants [51].

^f^According to the Flora of China [51] *C. botrys* is a synonym for *Dysphania botrys* (Linnaeus) Mosyakin & Clemants.

^g^Modern Tibetan *materia medica* and research data do not give the identification of this plant. Some treatises consider the term *kyap* as a synonym for *khur mang*.

^h^This botanical name is not mentioned in the Flora of China [51], yet it is regarded as a synonym for *A. jaquemontii* Kunth in the Flora of Pakistan [62].

^i^According to De’u dmar dge bshes [41] the term *gok nön* (*sgog sngon*) is a synonym for *ri gok* (*ri sgog*). dGa’ ba’i rdo rje [57] reports that the term *gok nön* is a synonym for *tsong gok* (*btsong sgog*), two types of which are distinguished: *gok tsong* (*sgog btsong*) and *gok tsong ri kye* (*sgog btsong ri skyes*, “*gok tsong* that grows on the mountains”). The former corresponds to *A. fistolosum*, cultivated as a vegetable since ancient times, the latter to *A. carolinianum*, which grows between 3,000 – 5,000 m. in west and north Tibetan Autonomous Region [51].

^j^Synonym for *Zanthoxylum oxyphyllum* Edgeworth [51].

## Author information

Alessandro Boesi is a Tibetologist. He obtained the Ph. D. at the Université de la Méditerranée (Marseille, France) in 2004. His thesis is entitled “Le savoir botanique des Tibétains: conception, classification et exploitation des plantes sauvages”. Since 1995 Alessandro has carried out research fieldwork in Himalayan and Tibetan regions investigating into the Tibetan concept of plant, and on the *materia medica* of Tibetan medicine. He has recently translated from Tibetan into Italian an ancient illustrated treatise on Tibetan medicinal plants (http://www.alessandroboesi.eu), and collaborates to a project on paper production and text printing in Tibet, conducted by a research team at the University of Cambridge (UK).
